# The pond snail *Lymnaea stagnalis*

**DOI:** 10.1186/s13227-020-00169-4

**Published:** 2020-12-04

**Authors:** Reiko Kuroda, Masanori Abe

**Affiliations:** Frontier Research Institute, Chubu University, 1200 Matsumoto-cho, Kasugai, Aichi 487-8501 Japan

**Keywords:** Chiromorphogenesis, Spiral cleavage, Learning/memory, Neurodegenerative diseases, Schistosomiasis, CRISPR/Cas9

## Abstract

The freshwater snail *Lymnaea stagnalis* has a long research history, but only relatively recently has it emerged as an attractive model organism to study molecular mechanisms in the areas of developmental biology and translational medicine such as learning/memory and neurodegenerative diseases. The species has the advantage of being a hermaphrodite and can both cross- and self-mate, which greatly facilitates genetic approaches. The establishment of body-handedness, or chiromorphogenesis, is a major topic of study, since chirality is evident in the shell coiling. Chirality is maternally inherited, and only recently a gene-editing approach identified the actin-related gene *Lsdia1* as the key handedness determinant. This short article reviews the natural habitat, life cycle, major research questions and interests, and experimental approaches.

## Natural habitat and life cycle

*Lymnaea stagnalis* is a freshwater snail, commonly known as the great pond snail. It belongs to the phylum Mollusca, class Gastropoda and family Lymnaeidae [1–3]. It is widely distributed in freshwater habitats over large parts of Europe, North America and Asia except its most southern region [4]. *L. stagnalis* prefers living in waters that flow slowly or in stagnant water bodies and occupies shallow pond margins with dense vegetation where it usually feeds on algae or decaying plants. It turns carnivorous at times and preys on newts and small-sized fish or its peer snails. It is a pulmonate and thus, in addition to the usual inhale/exhale oxygen from water, it breathes with its lungs by moving frequently to the surface to inhale air [3]. This trait allows adaptation to oxygen-poor environments.

**Fig. 1:**
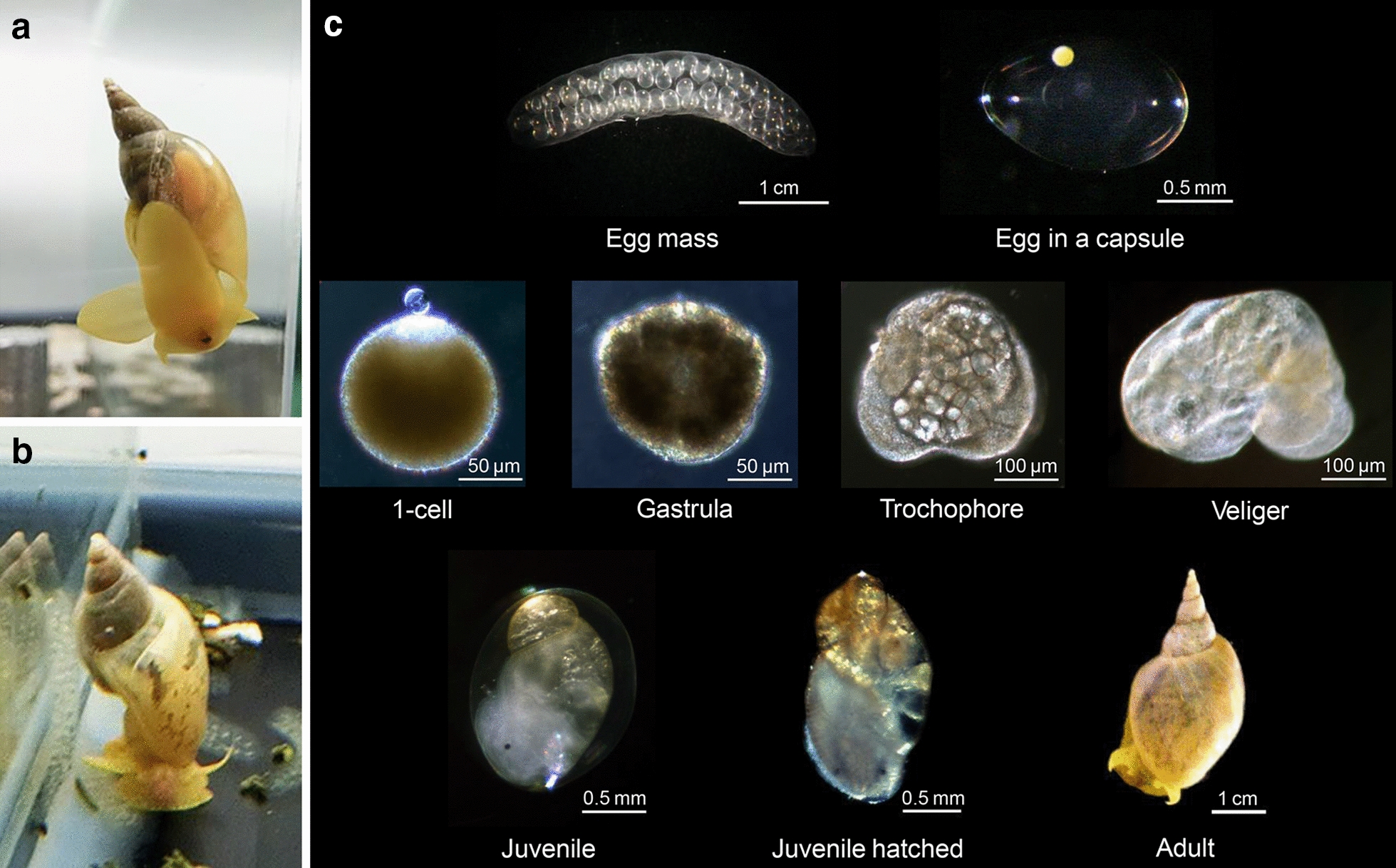
Adult snails and lifecycle. Sinistral (**a**) and dextral (**b**) snails reared in our group, and lifecycle showing images at the representative stages (**c**)

Although hermaphroditic, sexually mature *L. stagnalis* prefer cross-fertilization as is common in the freshwater pulmonates [5]. They can perform both female and male roles in mating. Copulation behavior and reproductive biology have been reviewed elsewhere [6, 7]. The snails lay eggs on weeds and other pond objects in large masses of about 2–6 cm, and which contain 50–120 eggs. Each egg, dark/intense yellow in color, measures about 100 μm in diameter, and is contained in an oval-shaped capsule (Fig. 1). Adult snails are 3–5 and 2–3 cm in shell length and width, respectively. Their size depends on the volume of water [3], with larger individuals found in large ponds [3]. The snail bodies are yellowish grey. Adults’ shells are yellow–brown in color, while immature/young snails have more translucent shells.

## Field collection and lab culture

Strains of *L. stagnalis* are kept in many laboratories around the world for various biological research purposes. One of the unique features of *L. stagnalis* is that it displays both coiling directions in the wild, 98% right-handed (dextral) and 2% left-handed coiling (sinistral) with the rare sinistral strains maintained by a few groups. We have established pure dextral and sinistral strains from snails kindly given to us by Dr. Guss Smit (Free University, The Netherlands), which we have been rearing for over 15 years [8]. They are maintained in water-circulating tanks, under a 16-h light/8-h darkness cycle, at a set temperature of 20–22 °C. They are fed with pet food for tropical fish and lettuce when young and mainly with lettuce after they grow up (Fig. 1a, b). The sizes of the laboratory-reared snails and their egg masses (and thus the number of egg capsules in each egg mass) are much smaller than those in the wild. Detailed procedures for maintaining adults and culturing embryos [1, 2] and characteristics of the early developmental stages have been described for *Lymnaeidae* [1–3]. *L. stagnalis* is quoted as living at least 1 year on average, sometimes longer, from 2 to 5 years [3]. Its lifespan is generally 6–12 months in our laboratory. Photographs of representative developmental stages are shown in Fig. 1c.

## Major interests and research questions

### Evolutionary aspects

*Lymnaea stagnalis* is one of the representatives of Spiralia, a morphologically diverse clade of protostome animals, including Mollusca (to which *L. stagnalis* belongs), Annelids, Platyhelminths and other taxa. Spiral cleavage is typically observed in this clade, but it is not unique to protostomes nor adopted by all members of the Spiralia [9, 10]. Thus, this group represents an excellent system for comparative studies to understand the origins of such diversity from a seemingly common ground plan [9]. With the advent of increasing genome sequence data and new molecular and functional experimental approaches in several species, spiralian phylogeny is now being revisited and reviewed [10].

### Chiromorphogenesis during development

Although most animals exhibit approximate anatomical bilateral symmetry externally, the internal organs display significant left–right asymmetry in terms of their shape and location. Mechanisms governing initiation and maintenance of this asymmetry are strictly controlled genetically. What, when, where and how is the chirality of the individual organism determined during development? Mechanisms underlying the left–right determination process have been intensively investigated in both vertebrates and invertebrates [11–15, and references therein]. Although similar signaling cascades are conserved among vertebrates and invertebrates, the onset of L–R establishment seems to vary among deuterostomes. The very recent work on *L. stagnalis* [16] is the first and still only the case in the animal kingdom to show at the molecular level that the handedness is already determined as early as the non-cleaved fertilized-egg stage.

**Fig. 2:**
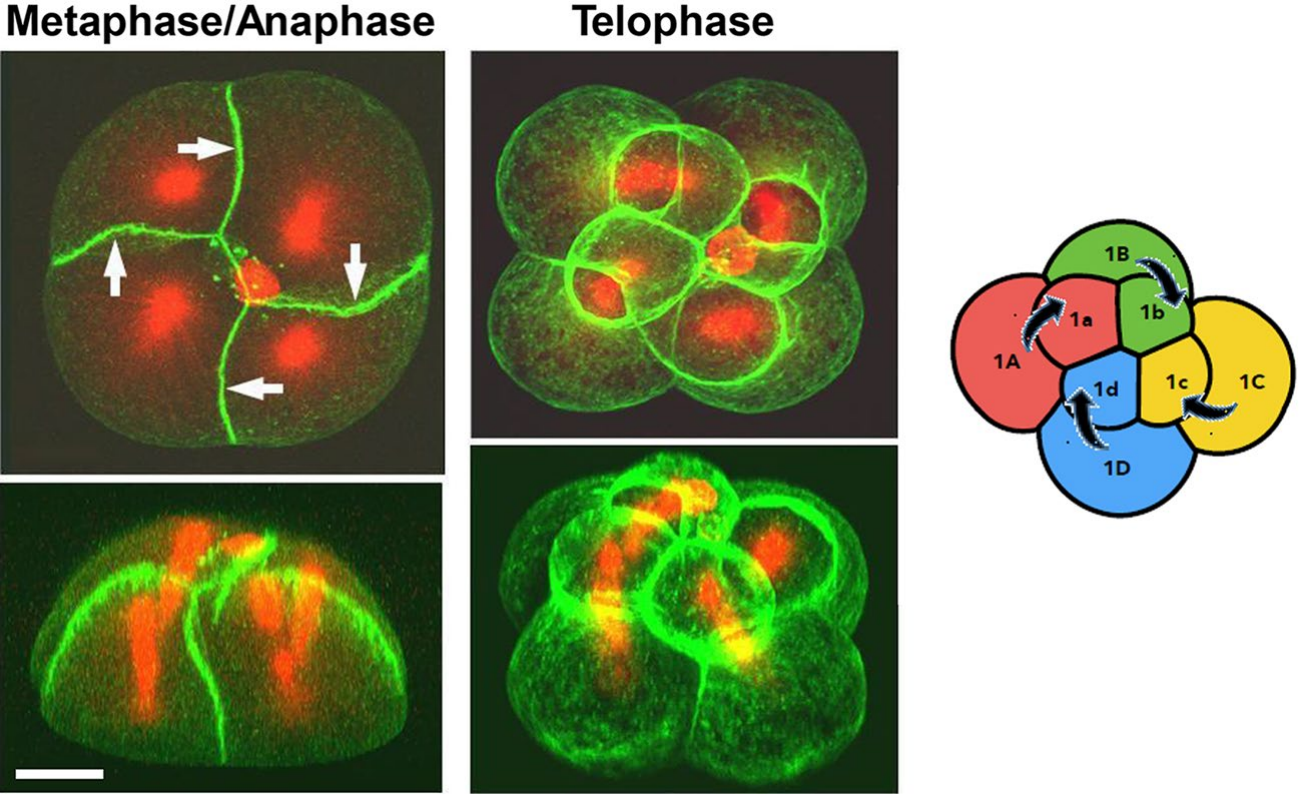
3D-reconstruction images of embryos in metaphase/anaphase and in telophase. Animal-view (top) and the corresponding lateral-view (bottom) images of embryos that are double-stained for filamentous actin (green, Alexa 488-phalloidin) and β-tubulin (red, Cy3-anti-β-tubulin antibody) of the dextral embryos. Arrows indicate SD (spiral deformation). Scale bar equals 20 μm. A schematic drawing to show the formation of micromeres from respective macromeres is shown. Confocal images were obtained with a laser scanning confocal microscope (Zeiss LSM 510). 3D-reconstruction images were made from a z-series of optical sections acquired every 0.80 μm. Adapted from [20]

*Lymnaea stagnalis* is an ideal target to answer these fundamental biological questions. Although genetic knowledge and experimental techniques for snails are limited compared with model animals such as *C.(Caenorhabditis) elegans* and *Drosophila*, *L. stagnalis* has unique advantages such as hermaphroditism, and a maternal mode of chirality inheritance [17]. The maternal mode of inheritance was proposed in 1923 for *Radix peregra* (previously known as *Lymnaea peregra*), an aquatic pulmonate gastropod in the same family *Lymnaeidae* [18, 19]. For *Lymnaea stagnalis*, chirality determination by a maternal single gene locus was indicated experimentally [15, 20, 21], and later decisively proven by genome editing [16]. The clockwise (CW) and anti-clockwise (ACW) micromere rotation at the 3rd cleavage (from the four- to the eight-cell stage) for the dextral and the sinistral embryos, respectively, was the earliest sign of chirality observed [22]. Asymmetric expression of *nodal/Pitx* genes was known to regulate asymmetric location/morphology of organs in vertebrates, and the genes were found to function in this snail as well [8, 16, 23, 24]. Surprisingly, the mechanical micro-manipulation of embryos at the third cleavage of *L. stagnalis* to reverse the rotation direction resulted in the expression sites of the *nodal/Pitx* genes at the mirror-imaged positions and produced healthy mirror-imaged animals (dextralized-sinistral and sinistralized-dextral snails). Their self-crossed offspring reverted naturally to the original handedness [8]. It is clear from this work that the relative location of the four micromeres and four macromeres at the 8-cell stage is definitive for handedness determination. During the course of the crucial third cleavage, the mirror symmetry relationship between the dominant dextral and recessive sinistral embryos is broken, as SD (spiral deformation) and SI (spindle inclination) are observed only for the dextral embryos (Fig. 2). Although these chiral cytoskeletal dynamics were shown to be strongly linked to the handedness-determining gene [17, 20, 25], they are auxiliary to give robustness to the chiral cell cleavage [8, 26]. Their loss does not change the chirality [8]. Chirality is determined by *lsdia1* already at the fertilized-cell stage and is firmly established at the eight-cell stage through the micromere–macromere contacts [8, 16]. These aspects are crucial to understanding the molecular process of handedness determination.

## Identification of the single handedness-determining gene

A maternal effect gene which is different between the sinistral and the dextral strains was initially identified as a candidate using positional cloning independently in 2016, by Davison et al. as “associated” [21] and by Kuroda et al. as “the strongest candidate” gene [15]. Neither study proved that the gene is in fact the handedness-determining gene. Kuroda et al. kept their gene naming of *Lsdia1* and *Lsdia2*, which correspond to Davison et al.’s *Ldia2* and *Ldia1*, respectively, because Davison et al.’s published gene and inferred protein sequences are different in key aspects from *Lsdia1/2* [26, 27]. There are tandemly repeated formin-related diaphanous genes, *Lsdia1* and *Lsdia2*, and a point mutation was found in both alleles of *Lsdia1* for the sinistral strains [15]. This abrogates expression of full-length LsDia1 protein, which is normally present already at the one-cell stage of the dextral embryos. No localization of *Lsdia1* nor *Lsdia2* mRNA was observed within a cell at the 1-cell stage, nor among blastomeres in the 2- or 4-cell stages [15, 26], although different results have been reported [21] (see “Experimental approaches” below for detail). Knocking out of *Lsdia1* in the dextral snail eggs using the CRISPR/Cas9 technique gave clear-cut results leading to the unequivocal identification of *Lsdia1* as the handedness-determining gene [16], which has been sought for nearly a century. Biallelic frameshift mutations introduced into the gene produced sinistrally coiled offspring generation after generation in the otherwise totally dextral genetic background. The gene sets the chirality already at the one-cell stage by twisting blastomeres either CW or ACW at the first cleavage, the earliest observed symmetry-breaking event linked directly to body-handedness in the animal kingdom [16]. The early intracellular chirality is superseded by the intercellular chirality during the third cleavage, leading to asymmetric expression of *nodal* and *Pitx* and then to organismal body-handedness [8]. Remarkably, all these characteristics at various developmental stages match with *Lsdia1* genotypes without exception, showing that the single gene dictates the handedness directly or indirectly across the biological hierarchy. This is the first successful germline transmission of a CRISPR/Cas9-edited gene in Mollusca [16, 26].

### Biomineralization

The molluscan shells have a broad diversity in terms of morphology, sizes, and ornamentations, as realized during the course of evolution. The molecular basis of the shell development is an intriguing and fundamental question. Shells consist of calcium carbonate and are typical examples of biominerals. In the area of shell development, *L. stagnalis* also serves as a model animal. The conserved early cell movements associated with initiation of shell construction have been observed [28]. The shells of gastropods have chirality, i.e., a spiral shape around a central axis. Asymmetric and mirror image patterns of the decapentaplegic (dpp) expression in the mantle edge between the dextral and sinistral lineages of *L. stagnalis* have been reported [29]. More recently, relevant asymmetrically expressed molluscan shell matrix proteins (SMPs) were found using proteomic and transcriptomic datasets in the left and right sides of mantle tissue [30, 31]. Recent exciting methodological developments available to the molecular biologist open a new channel for communication between biologists and mineralogists with common interests in a variety of aspects of biomineralization, ranging from structural biology to evo-devo, to material properties and beyond [32, 33].

### Neuroscience

The relatively simple central nervous system (CNS) of *Lymnaea*, with its large and identifiable neurons, has facilitated its adoption as a major model in neurophysiology and psychology research, for learning and memory studies [34]. The neurons are accessible for detailed electrophysiological, biophysical, biochemical, and molecular studies [35–37]. Unlike *D. melanogaster* and *C. elegans* which are the most common and best characterized invertebrate models, *Lymnaea* has a relatively long life span which allows the study of age-related modifications involving genetic, molecular, and cellular mechanisms, and which usually take time to manifest their full effects [38, 39]. Using food-reward classical conditioning experiments, the crosstalk between neuronal metabolism and the formation and the maintenance of long-term memory and how such mechanisms are altered during ageing have been investigated. For example, insulin and IGF-1 [40–43], NO-cGMP signaling [44] and CREB [45] have been reported to modulate aspects of plasticity in the CNS of *Lymnaea* and enhance learning abilities in older learning-impaired snails. These findings resonate well with the growing evidence suggesting a role for insulin-like peptides and insulin resistance in human ageing [46, 47]. The CNS of adult *L. stagnalis* is capable of spontaneous regeneration following neuronal injury. Thus, *L. stagnalis* could serve as a valuable animal model in which to study the cellular mechanisms underlying neuronal regeneration [48, 49].

*Lymnaea* also provides an attractive platform to investigate human neurodegenerative disorders such as Alzheimer’s and Parkinson’s [42, 50]. Several genes relevant in aging and neurodegenerative/other diseases were found to be evolutionary conserved in *L. stagnalis* [50], and a direct link between administration of β-amyloid (Aβ) and loss of consolidated LTM (long-term memory) was observed in *L. stagnalis* as in humans [51]. In addition to the relatively long lifespan, the animal has another great advantage for neuroscience research, namely it lacks a blood–brain barrier [51]. Consequently, for example in dementia studies, it is not necessary to apply Aβ directly to the brain tissue. Thus, the Aβ concentration can be kept low and well controlled for each individual animal. Aβ-induced memory loss and electrophysiological changes can be studied in the absence of neuronal death in a defined network underlying associative memory. Interestingly, both the behavioral and neuronal effects were reported to depend upon the animals having been classically conditioned prior to treatment, since Aβ application before training caused neither memory impairment nor underlying neuronal changes over a comparable time period [51].

*Lymnaea stagnalis* may offer to translational medicine a powerful new tool to study age-related diseases of the nervous system by identifying new molecular targets for the development of innovative therapeutic strategies, and by enabling the screening of large numbers of compounds for drug activity. Moreover, the snail system does not have the serious ethical and economic issues associated with the animal models currently most frequently used in screening, i.e., rats, mice and primates [39].

### Schistosomiasis

**Fig. 3:**
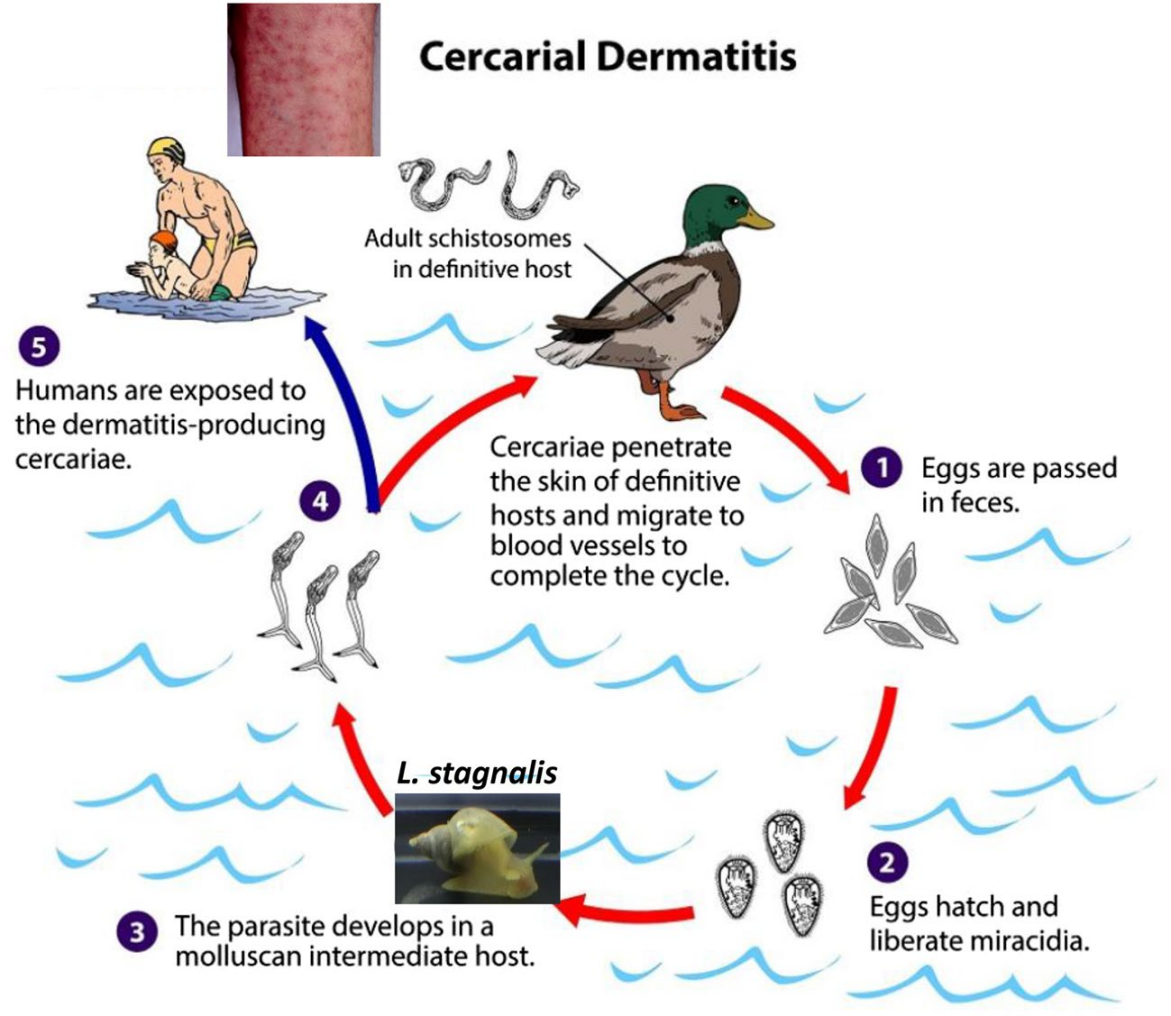
Life cycle of parasite avian schistosome *Trichobilharzia szidati* which involves *L. stagnalis* as an intermediate host.: Cercarial dermatitis in humans is caused by the parasite. Adapted and modified from https://www.cdc.gov/parasites/swimmersitch/biology.html)

*Lymnaea stagnalis* serves as the intermediate host for more than one hundred species of digenetic trematodes, including the avian schistosome *Trichobilharzia szidati*, a causative agent of cercarial dermatitis in humans (Fig. 3) [52]. A more serious disease is human schistosomiasis caused by several different parasites including *Schistosoma mansoni* for which the freshwater snail *Biomphalaria (B.) glabrata* is the specific intermediate host. Schistosomiasis continues to affect the health of 220 million people around the world. The World Health Organization lists schistosomiasis as one of the “Neglected Tropical Diseases (NTDs)” [53]. Considerable effort has been invested over several decades to understand the immunological responses of *B. glabrata* to various microorganisms. As a consequence, a large number of immune- and stress-responsive genes and gene products have been documented, but most of them need to be functionally verified [54]. As *B. glabrata* and *L. stagnalis* are phylogenetically closely related, the recent application of CRISPR–Cas9-mediated genome editing to *Lymnaea* [16] should allow functional characterization of these immune-related genes [55, 56].

### Ecotoxicology

*Lymnaea stagnalis* is also a popular model organism for studies of ecotoxicology [57, 58] and a bioindicator of aquatic contaminants [57]. Toxicity studies using *L. stagnalis* began to appear in the late the 1970s, however, it is after the late 1990s that a great number of toxicological investigations focused on the sensitivity to metals, e.g., aluminum, mercury, cadmium, and particularly to lead [59]. In recent years, several works addressing the environmental risk assessment of chemicals (tributyltin and coal tar) and micro-plastics, were published also using this snail [60–62]. *L. stagnalis* has been accepted as a standard test organism for ecotoxicological studies with an OECD guideline for a reproduction test (OECD, 2016).

## Experimental approaches

### Genetics

In *Lymnaea*, the forward genetics approach has been used to identify candidate genes including the gene determining left–right shell coiling [15–19, 21] (see “Chiromorphogenesis during development” section). We have constructed F10 congenic lines by continuous backcrossing and searched for recombinant individuals [15, 17]. These were possible as *L. stagnalis* is prolific, however, creating linkage markers was not easy due to lack of an annotated genome database. Thus far, the methods using AFLP (amplified fragment length polymorphism) and RAD (restriction-site associated DNA) markers have been effective [15, 63]. We constructed a BAC library of the right coiling strain and sequenced the handedness-determining locus by chromosome walking [15], but mapping using draft genomic data [21] may also be possible.

### Embryological manipulations

For many experimental protocols, embryos must be taken out of the egg capsule prior to various treatments. Egg mass is collected from the aquarium maintaining the adult snails, and the egg capsules are isolated by rolling the egg mass on a sheet of filter paper to remove the surrounding jelly. Egg capsules are cultured in 1.5 × HF (Holtfretter’s) solution [16]. Eggs can be easily removed from the capsule using tweezers, and cultured in 5 × HF solution.

**Fig. 4:**
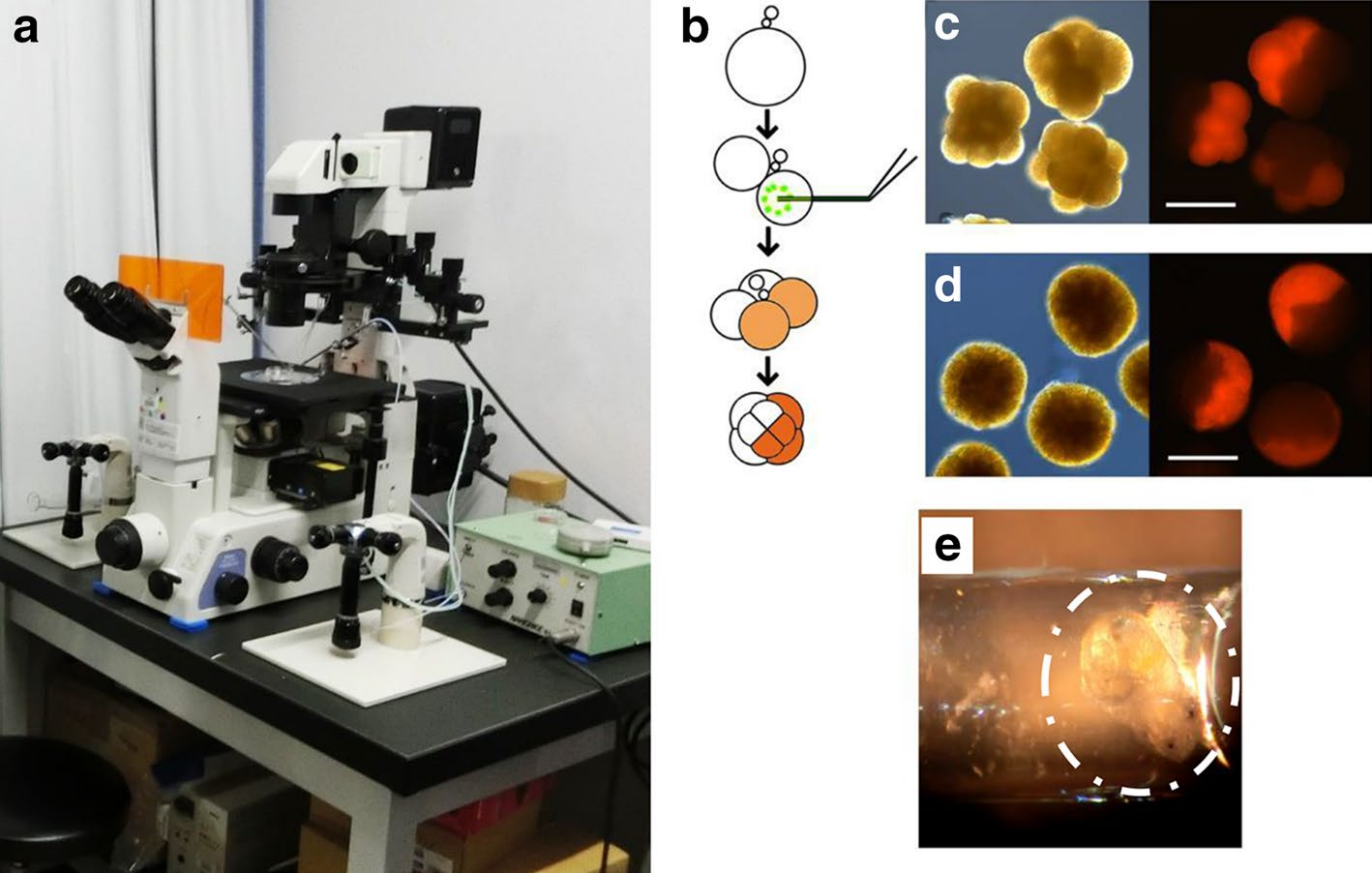
Injection of fluorescent protein mRNA for cell-lineage tracing in *L. stagnalis* embryos. Standard setup for microinjection (**a**), schematic diagram of the experiment showing an injection to one cell at the two-cell stage (**b**), and fluorescent images of embryos where half of the cells are expressing DsRed-Express at the 8-cell (c, right panel) and blastula (d, right panel) stages; bright-field images (c and d, left panel) are also included. The exposure time for the fluorescence image capture was 4 s (**c**) or 2 s (**d**). Scale bar 100 μm. For other types of experiments, embryos after microinjection were cultured in a capillary tube. 4e shows a juvenile snail (encircled) cultured inside a capillary. **b**–**d** are adapted from [64]

Microinjection of a liquid into early *L. stagnalis* embryos is not easy but possible. Eggs are pretreated with dithiothreitol (DTT) for a short period of time before microinjection in order to weaken the tough vitellin egg membrane. Careful DTT treatment is essential, as developmental abnormalities are often observed when embryos experience prolonged DTT treatment. The eggs are transferred to a droplet of injection buffer on the sample stage. A micropipette is filled with relevant reagents such as mRNAs in nuclease-free water with Lucifer yellow as a marker dye and forced into embryos rapidly by positive pressure with an injector (Fig. 4a). In the authors’ laboratory, microinjections are performed using a micromanipulator (MN-4, Narishige), motor-drive microinjector (IM-30, Narishige) and inverted fluorescent microscope (Nikon TE300) (Fig. 4a). After the injection, embryos are transferred into glass capillary tubes and are cultured until they develop into juvenile snails (Fig. 4e). Juveniles are transferred to small aquaria and reared to adults. Details of embryo manipulations have been published in Refs [8, 15, 64].

### Whole-mount in situ hybridization

Whole-mount in situ hybridization (WISH) is a common technique used for visualizing the location of expressed RNAs in embryos. Based on protocols for the sea water snail *Patella vulgata* [65], those for *L. stagnalis* have been developed to discriminate RNAs even from genes exhibiting 89.4% sequence identity [8, 15]. Appropriate controls must be carried out to avoid misleading conclusions caused by artifacts. For example, caution should be exercised with ‘within-capsule’ fixing procedures (in which the embryo is not removed from its capsule), with which remarkable asymmetric localization for the transcripts of housekeeping genes, β-actin and β-tubulin, was observed [26], just like the asymmetric expression of *dia* genes in the literature [21]. Standard outside capsule protocols, on the other hand, do not give any localization for β-actin, β-tubulin, *Lsdia1* nor *Lsdia2* genes [15, 25]. β-tubulin showed the homogeneous presence of the mRNA from the 1-cell stage before the first polar body extrusion [15], although it was previously reported undetected by WISH [66].

### Immuno-staining and Western blotting

Immunostaining has been applied to visualize spindle architecture in the sea water snail *Illyanassa* using fluorophore-conjugated anti-β-tubulin antibodies [67]. Figure 2 shows representative images of double staining of filamentous actin and microtubules of *L. stagnalis* [20]. Western blot analyses for *L. stagnalis* clearly showed that LsDia1 protein is present in the dextral embryos from the 1-cell stage immediately after oviposition to the blastula stage, but is not detectable at any stage for the sinistral embryos [15]. For these experiments, 50–100 embryos of similar developmental stage are needed to provide sufficient extract for analysis.

### Drug inhibition

Drug inhibition experiments can provide information on the functions of target proteins. For most experiments on *L. stagnalis*, drugs can be applied to eggs within the capsules. However, if timing is critical and a delay of drug delivery to the capsules and embryos does matter, then decapsulated eggs should be used. If inhibitor drug is not easily soluble in aqueous solution, DMSO can be added up to 0.3% concentration without apparent effect on *L. stagnalis* [15, 20]. Inhibition of actin or tubulin polymerization by latrunculin A and nocodazole, respectively, revealed that SD and SI are introduced by actin and not by microtubules [20]. Similarly, GSK3β inhibition by the highly specific 1-azakenpaullone (AZ) and by LiCl revealed a short sensitive period from the 2- to 4-cell stage which induces a subsequent dramatic developmental delay and alteration of the cleavage patterns of blastomeres at the fifth cleavage (16- to 24-cell stage) [68].

In general, it is important to check the drug’s specificity and the drug or solvent lethality. Two independent inhibition studies on *L. stagnalis* early embryos using the anti-formin drug SMIFH2 gave different conclusions. One showed 100% lethality at concentrations higher than 10 µM regardless of the timing of drug application [15, 25], whereas the other used 100 µM [21, 26]. Statistics should be checked on the ratio of dead/tested embryos in comparison with control experiments to avoid focusing on a rarely observed phenotype. A similar point was raised about drug inhibition experiments on the freshwater gastropods *B. glabrata* and *L. stagnalis* using Dorsomorphin and SB431542, respectively. In both cases, it was found that the main impact on gastropod embryogenesis was lethality and not phenotypic changes to shell development [69].

### Overexpression and knock-down

In vitro-synthesized mRNA expression is important for the understanding of molecular mechanisms during development. Expression of in vitro-synthesized mRNAs in *Lymnaea stagnalis* was shown to be possible for the first time by micro-injecting the mRNAs of fluorescent proteins, mCherry, DsRed-Express, and enhanced green fluorescent protein into the eggs before the first polar body stage. They are expressed and fluorescence was detected within a few hours of injection [64] (Fig. 4b–d). The distribution of β-Catenin in vivo by micro-injecting GFP-tagged β-catenin [70], live F-actin using a GFP fusion of the actin-binding domain of utrophin and live microtubules using GFP or RFP fusions of the MT binding domain of ensconsin [71] were followed in the seawater snail *Crepidula fornicata*.

In *L. stagnalis*, RNAi knock-down experiments have been reported in adult snails that disrupt neuronal nitric oxide synthase gene function [72]. As functional analysis by morpholino was successful in sea snails [70, 73], this method may also be applicable to *L. stagnalis*.

### CRISPR/Cas9-mediated gene editing

CRISPR/Cas9 genome editing allows for a more targeted analysis of gene function [74]. This technique has been applied to a variety of organisms including non-model animals, however, mosaicism is the serious obstacle at F0, particularly when the method is used in embryos [16, 75, 76]. Functional analyses must be carried out at F1 where homogeneity in somatic cells of the whole body is realized. In this regard, hermaphroditism and the ability to perform both cross- and self-mating in *L. stagnalis* are a great advantage for CRISPR work. Specifically, homozygous and heterozygous knockout F1 snails can be obtained by self-crossing of a F0 snail, which was microinjected with Cas9 mRNA and guide RNA at the one-cell stage in a manner described in Embryological Manipulations. Self-fertilization of heterozygous knockout F1 can establish F2 of particular genotype with the otherwise identical genetic background. These lines can be retained generation after generation [16]. In the total of four experiments, 39 injected embryos were cultured. Ten F0 adult snails were obtained (26%), five of which showed germline transmission to the F1 generation [16]. Practice is needed for successful microinjection (Fig. 4a, b) and exo ovo culturing (Fig. 4e) of embryos [8, 16], however, the most demanding part may be the individual breeding and rearing of many snails.

Transgenic snails have not yet been produced, although several techniques have been developed in mollusca but not yet used for functional assays [77–79]. The possibility to generate a knock-in snail line using the CRISPR technique was indicated for a sea water snail, where transient transgenic expression was achieved [80]. This may be a future direction to pursue, to enable live imaging of target gene products.

## Research community and resources

MolluscDB [81], a GenomeHubs database for Mollusca, has been built and the STAGIG genome sequencing project for *L. stagnalis* has been launched. An early version of the data has been published [21] and publication of the annotated sequence data is eagerly awaited. There is a *Lymnaea stagnalis* Sequencing Consortium web site (http://www.lymnaea.org/members.html). Table 1 summarizes the latest data bases.

**Table 1:** Resource/database available for *L. stagnalis* research

	Pond snail, tissue	Description/sequencing technology	References
Genome	*Lymnaea stagnalis* (Lymnaeidae)	De novo draft assembly/Illumina	[21]
	*Radix auricularia* (Lymnaeidae)	De novo draft assembly/Illumina	[82]
	*Biomphalaria glabrata* (Planorboidea)	De novo draft assembly/Sanger, Roche454, Illumina	[83]
Transcriptome	CNS	1320 ESTs/Sanger	[84]
	CNS	7712 ESTs/Sanger	[36]
	CNS	116,355 TSAs/Illumina	[85]
	Adult tissues	Gene expression response to drug/Roche 454	[86]
Transcriptome and proteome	Adult tissues, larval stages	34 shell forming candidates/Illumina	[30]
	Mantle	L–R asymmetric expression/Illumina	[31]
Small RNA	Reproductive tract, foot	Small RNA sequence/BGISEQ	[87]

## Data Availability

Not applicable.

## Abbreviations

GSK3β: Glycogen synthase kinase 3β
DMSO: Dimethyl sulfoxide
GFP: Green fluorescent protein
RFP: Red fluorescent protein
NO: Nitric oxide
cGMP: Guanosine 3′,5′-cyclic monophosphate
CREB: Cyclic AMP-responsive element binding protein
CNS: Central nervous system
